# Transcriptomic Analysis of *Staphylococcus epidermidis* Biofilm-Released Cells upon Interaction with Human Blood Circulating Immune Cells and Soluble Factors

**DOI:** 10.3389/fmicb.2016.01143

**Published:** 2016-07-21

**Authors:** Angela França, Gerald B. Pier, Manuel Vilanova, Nuno Cerca

**Affiliations:** ^1^Laboratory of Research in Biofilms Rosário Oliveira, Centre of Biological Engineering, University of MinhoBraga, Portugal; ^2^Division of Infectious Diseases, Department of Medicine, Brigham and Women's Hospital/Harvard Medical SchoolBoston, MA, USA; ^3^Instituto de Ciências Biomédicas de Abel Salazar, Universidade do PortoPorto, Portugal; ^4^Instituto de Investigação e Inovação em Saúde, Universidade do PortoPorto, Portugal; ^5^Instituto de Biologia Molecular e Celular, Universidade do PortoPorto, Portugal

**Keywords:** *Staphylococcus epidermidis* biofilms, biofilm-released cells, human blood, human plasma, human leukocytes, transcriptome

## Background

The colonization of indwelling medical devices by biofilm-forming bacteria is one of the major causes of healthcare-associated infections (Percival et al., 2015). *Staphylococcus epidermidis*, a biofilm-forming commensal bacterium that inhabits human skin and mucosae, is considered one of most important causes of medical devices-related infections, being particularly associated with the use of intravascular catheters (Mack et al., 2013). Although *S. epidermidis* biofilms are classically associated with the development of chronic infections (Costerton et al., 1999), the release of cells from the biofilm has been associated with onset of acute infections such as embolic events of endocarditis (Pitz et al., 2011), bacteremia, or even septicemia (Cole et al., 2016). Bloodstream infections caused by *S. epidermidis* are typically indolent and difficult to eradicate significantly increasing patient's morbidity (Kleinschmidt et al., 2015) and mortality among immunocompromised (Khashu et al., 2006) and immunosuppressed patients (Bender and Hughes, 1980). In addition, the costs associated with the diagnosis and treatment of these secondary infections is estimated to be approximately $20,000 per occurrence (Kilgore and Brossette, 2008). Henceforth, it is imperative to redefine strategies for the management of the pathologic events associated with biofilm disassembly. Since bloodstream infections are one of the most frequent complications caused by *S. epidermidis* biofilm disassembly (Cole et al., 2016), a comprehensive analysis of the interplay between *S. epidermidis* biofilm-released cells (BRC) and hosts' blood components would be invaluable. Herein, as the first step toward the understanding of this interaction, we have characterized, using RNA sequencing (RNAseq) technology, the transcriptome of *S. epidermidis* BRC upon interaction with whole human blood, polymorphonuclear, or mononuclear leukocytes and plasma.

## Materials and methods

### Ethics statement

Human blood was collected from healthy adult volunteers, under a human subject's protocol approved by the Institutional Review Board of the University of Minho (SECVS 002/2014). Furthermore, this procedure was performed in agreement with Helsinki declaration and Oviedo convention. All donors gave written consent before blood collection.

### Bacteria and growth conditions

*S. epidermidis* strain 9142, isolated from a blood culture (Mack et al., 1992), was used for this study. BRC were obtained using a fed-batch system in the presence of Tryptic Soy Broth (TSB) supplemented with 0.65% glucose and under agitation conditions, as detailed elsewhere (França et al., 2016). BRC cells were collected from 12 different originating biofilms and pooled together to decrease the variability inherent to biofilm growth (Sousa et al., 2014). After 10 s sonication at 33% amplitude (Cole-Parmer 750-Watt Ultrasonic Homogenizer 230 VAC, IL, USA), the concentration of BRC was adjusted to 1 × 10^9^ total cells/mL, by flow cytometry (EC800, Sony Biotechnology Inc., CA, USA), using SYBR Green (Invitrogen, CA, USA) and propidium iodide (Sigma, MO, USA) staining as previously optimized (Cerca et al., 2011).

### Blood collection and fractioning

Peripheral blood was collected into BD Vacutainer® tubes coated with lithium heparin (BD®, NJ, USA). Plasma was separated from the cellular fraction by centrifuging whole blood at 1440 g for 20 min at 4°C. Mononuclear (MN) leukocytes were purified from whole blood using Histopaque 1077 gradient (Sigma) as indicated by the manufacturer. Thereafter, the mononuclear cells-depleted pellet resultant from the Histopaque 1077 gradient was incubated with 1.5% (v/v) dextran solution during 35 min, at room temperature, in order to separate polymorphonuclear (PMN) cells from erythrocytes. PMN cells (present in the supernatant) were then transferred in to a new tube and harvested by centrifugation at 450 g for 15 min at 4°C. Both PMN and MN cells were incubated with water for 30 s to lyse the remaining erythrocytes and, after readjusting the isotonic conditions by adding 10 × PBS, leukocytes were collected by centrifugation at 200 g for 15 min at 4°C. Finally, leukocytes were suspended in 0.5 mL of donor's plasma and samples purity and viability determined by flow cytometry (EC800, Sony) using, respectively, CD15 (PMN) and CD3 (MN) (eBioscience, CA, USA) and propidium iodide staining (5 μg/mL, Sigma). Only samples with purity ≥90% and death ≤ 15% were used. The number of PMN and MN cells was determined also by flow cytometry and the concentration adjusted, in donor's plasma, to 1.0 × 10^6^ cells/mL.

### Co-incubation of bacteria with whole human blood and its circulating immune factors

In 2 mL tubes, 100 μL of a suspension of 1 × 10^9^ total BRC/mL were mixed with 900 μL of whole human blood, PMN, or MN at 1.0 × 10^6^ cells/mL, plasma or TSB (containing the same concentration of heparin as blood and its components) and incubated at 80 rpm (in a 10 mm orbit incubator), for 2 h at 37°C. After the co-incubation period, samples were sonicated for 5 s at 33% amplitude (Cole-Parmer) in order to lyse, and thus, decrease eukaryotic cells contamination. Finally, bacteria were harvested by 5 min centrifugation at 16,000 g at 4°C and immediately suspended in 1 mL of RNA protect™ bacteria reagent (QIAGEN, Hilden, Germany), which was diluted 2:1 in nuclease-free water (Gibco, MD, USA). BRC before the co-incubation assays (T0h) were also collected. This assay was performed four independent times using blood of four different donors (both female and male).

### RNA isolation and libraries construction for RNA sequencing analysis

Total RNA was isolated using RNeasy mini kit (QIAGEN). Bacterial cell lysis was achieved by mechanical (3.0 mm zirconium beads, Sigma) and chemical lysis (phenol, Fisher Scientific, MA, USA) as optimized before (França et al., 2012). RNA quality was assessed using an Experion™ automated electrophoresis system (Bio-Rad, CA, USA). RNA quality indicators were above 9 for all samples. Total RNA purified from each of the four independent co-incubation assays performed were pooled together to decrease donor-associated variability. Subsequently, pooled RNA was treated with TURBO DNase (Ambion, NY, USA) and acid-phenol:chloroform:isoamyl alcohol (125:24:1) (Ambion, MA, USA) to degrade and isolate, respectively, contaminating genomic DNA. Potential contaminating eukaryotic RNA was removed using MICROB*Enrich*™ kit (Ambion). Prokaryotic messenger RNA was then enriched by depleting ribosomal RNA using Ribo-Zero™ rRNA removal kit for Gram-positive bacteria (Illumina, CA, USA) and transcriptomic libraries were constructed using ScriptSeq™ RNA-seq library preparation kit (Illumina). The quality of the libraries constructed for each condition under study was assessed by quantitative PCR and Hi-Sensitivity D1K TapeStation (Agilent 2200 TapeStation). Finally, all libraries were multiplexed and sequencing data was generated in a MiSeq® sequencer (Illumina) from paired-end reads (2 × 150 bp).

The results obtained were validated by quantitative (q) PCR, as described earlier (Sousa et al., 2014). Total RNA samples utilized for RNAseq analysis were used as template. The sequences of the primers used are shown in Supplementary Table 1. Fold-change values were determined applying the Pfaffl method (Pfaffl, 2004) using T0h as control.

### Trimming and data analysis

After sequencing, adapters were trimmed by MiSeq® internal software during the base calling. CLC Genomics Workbench version 5.1 (QIAGEN) was then used for quality, ambiguity, and length trimming, alignment with *S. epidermidis* RP62A (GenBank accession number: CP000029.1), normalization of the reads *per* kilobase *per* million mapped reads (Mortazavi et al., 2008), and for the analysis of differential gene expression. Quality, ambiguity, and length trimming were performed using the CLC genomics workbench default settings. The transcriptomic alterations induced on BRC incubated with human blood or its circulating immune cells and soluble factors was determined using as control BRC transcriptomic profile before co-incubation assays. Kal's statistical test (Kal et al., 1999), with false discovery rate (FDR) (Pawitan et al., 2005), was applied to identify statistically significant alterations in gene transcription. Alterations with fold changes below 3 and *P* values above 0.05 were discarded. Taking into consideration that bacteria may change their transcriptome, in a nonspecific manner, after being transferred in to a new environment, we had also compared the transcriptomic alterations observed in BRC incubated with whole blood or its cellular and soluble components with those observed in the presence of TSB, a standard laboratorial medium. Hence, in order to only consider alterations induced by biological factors, genes whose transcription was found in both groups were discarded. Nevertheless, genes with opposite regulation in both groups were maintained. KEGG analysis was performed using STRING (Franceschini et al., 2013) and only gene-sets passing significance thresholds (*P* < 0.05, Hypergeometric test with FDR) are depicted. The heat map was created using CIMminer utilizing Euclidean distance method and average linkage clustering.

## Data deposition

The transcriptomic profile of *S. epidermidis* BRC under the conditions described in this study was deposited in Gene Expression Omnibus (GEO) database, at NCBI, under the accession number GSE79948. Raw and trimmed/filtered datasets are available and can be downloaded as fastq and text files, respectively.

## Interpretation of data sets

Bacteremia is one of the major clinical complications associated with the release of cells from *S. epidermidis* biofilms formed on intravascular catheters. These infections are associated with increased hospitalization periods, healthcare costs and patient morbidity, or even mortality (Kleinschmidt et al., 2015). Our goal was thus to gather the first insights into the interaction between *S. epidermidis* BRC and human blood, in order to better understand the role of these cells in the pathogenesis of *S. epidermidis* biofilm-related infections. To this end, we analyzed the transcriptome of BRC upon interaction with human blood or its cellular and soluble components, using an *ex vivo* model previously used for other microorganisms (Fradin et al., 2003; Mereghetti et al., 2008; Malachowa et al., 2011; França et al., 2014).

**Figure 1:**
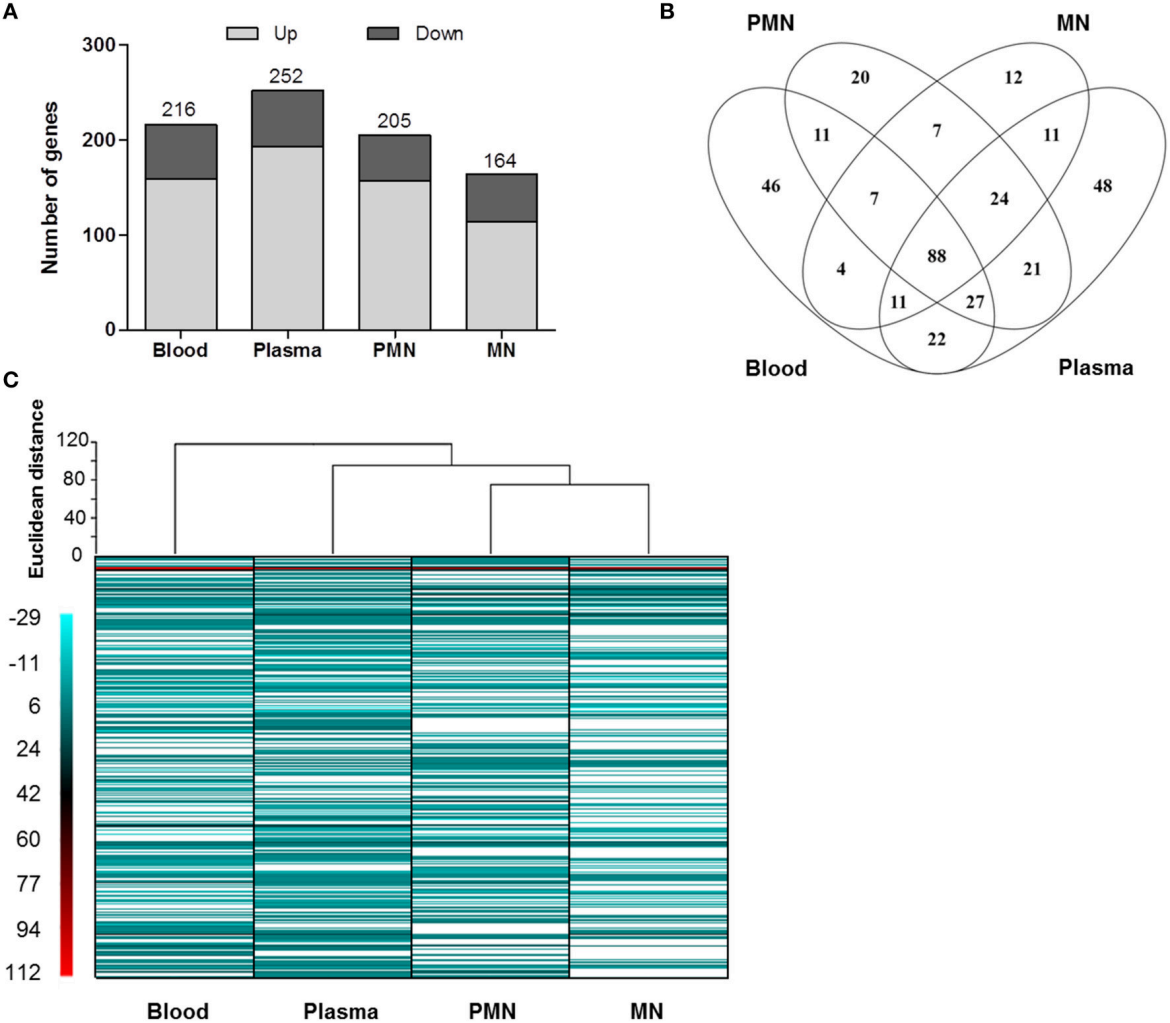
**Overview of the significant alterations in the gene transcription profile of BRC incubated with human blood or its cellular and soluble components**. **(A)** Number of genes with increased and decreased transcription; **(B)** Venn diagram showing the number of genes uniquely (disjointed) and commonly expressed (overlapping) among the conditions tested; **(C)** Heat map showing the expression pattern as well as hierarchical clustering of all samples. The white bands represent genes whose expression was not significantly altered (*P* > 0.05 and fold change < 3). PMN: polymorphonuclear leukocytes, MN: mononuclear leukocytes.

The transcriptome of *S. epidermidis* BRC changed significantly after incubation with whole human blood or its cellular or soluble components. Nevertheless, before analyzing the alterations observed, RNAseq results were confirmed using qPCR. As can be seen in Supplementary Figure 1, a strong correlation between both methods was observed. Thereafter, we compared the transcriptome of BRC incubated with human blood, PMNs, MNs, or plasma with the one of BRC incubated with TSB, a rich medium frequently used in *in vitro* assays. This comparison allowed the identification the genes whose transcription was affected only by the presence of biological factors. Within the genes found differentially transcribed (fold change >3 and *P* < 0.05, Kal's test with FDR) only 21–25% of the genes were found in BRC incubated with whole human blood or its cellular and soluble components and these were the genes considered for further analysis (Figure 1).

**Figure 2:**
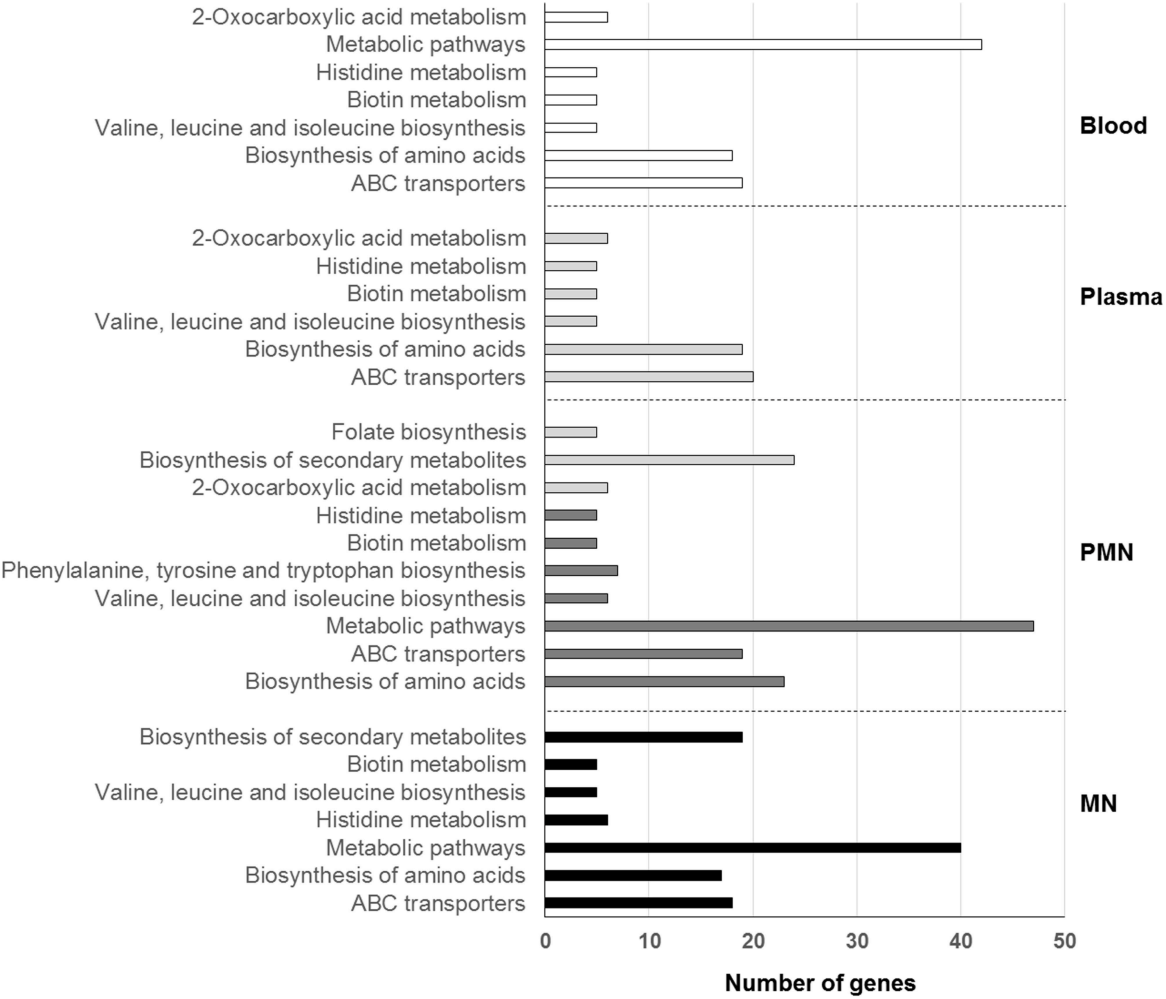
**KEGG pathways enriched after incubation with whole human blood or its cellular or soluble components**. KEEG pathways are organized, for each condition, from lower (top) to higher (bottom) significance. PMN, polymorphonuclear leukocytes; MN, mononuclear leukocytes.

KEGG pathways analysis showed that the major alterations occurring in the presence of human blood circulating factors were associated with basic pathways involved in the biosynthesis or metabolism of amino acids and in the import or export of substances (Figure 2). In addition, KEGG pathways such as biosynthesis of secondary metabolites and phenylalanine, tyrosine, tryptophan, and folate biosynthesis were also found significantly enriched (*P* < 0.05, Hypergeometric test with FDR) but only in BRC incubated with both PMN and MN leukocytes (Figure 2).

As it would be expected in a low free iron environment such as human blood/plasma, iron transferrin receptors, iron ABC transporter permeases, and iron ABC transporter ATP binding proteins were found significantly enriched. Interestingly, biotin metabolism was also found significantly enriched (*P* < 0.05, Hypergeometric test with FDR) in all the conditions tested. In fact, the complete biotin operon, which is composed by the genes *bioA, bioD, bioF*, and *bioW*, was found highly transcribed, with fold change values ranging from 13 to 110. The same observation was made in *S. aureus* after short-term incubation with either blood or plasma (Malachowa et al., 2011). Biotin is an important cofactor involved in prokaryotic central pathways being particularly important during infection, as bacteria have a high demand for micronutrients (Streit and Entcheva, 2003). Due to the essential nature of biotin metabolism, the target of biotin metabolism-associated proteins has been under special attention, being considered a promising strategy to combat drug-resistant pathogens including *S. aureus* (Soares da Costa et al., 2012; Pendini et al., 2013; Paparella et al., 2014). No enrichment was found among genes with decreased transcription. Interestingly, in all the conditions tested, ~40% of the genes with down-regulated transcription encoded hypothetical proteins or pseudogenes.

Analyzing the effect of each of the blood components independently, using hierarchical cluster analysis, we found two major clades: one that separates BRC incubated with whole human blood from the remaining conditions and a second clade that separates BRC incubated with plasma from those incubated with either PMN or MN cells (Figure 2). Interestingly, this suggested that both PMN and MN leukocytes, despite their involvement in innate immunity and phagocytic capability, had little influence on *S. epidermidis* BRC gene expression profile. Also, these results indicated that the majority of the alterations observed in whole human blood occurred in plasma, as recently observed in a small panel of *S. epidermidis* genes (França and Cerca, 2016). We cannot, however, discard the possibility that a different effect on *S. epidermidis* gene expression profile could be seen if these cells were circulating in human blood, as crosstalk with and production of signaling molecules by other cells may have an important influence in *S. epidermidis* gene expression.

## Conclusions

Overall, this study enabled us to identify the pathways involved in the adaptation of the bacterium to the stressful environment encountered in human blood consequently contributing to its survival and persistence. Hence, these results may be helpful in the selection of potential targets for future studies aiming to develop preventive and/or therapeutic strategies for *S. epidermidis* biofilm-based infections. One interesting target may be biotin metabolism-associated proteins, which are already being tackled in other important human pathogens.

## Author contributions

GP, MV, and NC conceived the study and participated in its design and coordination. AF performed the experiments, collected, analyzed, and deposited the data. AF prepared the draft and GP, MV, and NC proofread the final draft. All authors have read and approved the manuscript.

## Funding

This study was funded by the Portuguese Foundation for Science and Technology (FCT) by the project with the reference FCOMP-01-012014-FEDER-041246 (EXPL/BIA-MIC/0101/2013), the strategic funding of UID/BIO/04469/2013 unit, COMPETE 2020 (POCI-01-0145-FEDER-006684), BioTecNorte operation (NORTE-01-0145-FEDER-000004) funded by European Regional Development Fund under the scope of Norte2020 - Programa Operacional Regional do Norte. NC is an Investigador FCT. AF is supported by the FCT fellowship SFRH/BPD/99961/2014. The funders had no role in study design, data collection and interpretation, or decision to submit the work for publication.

### Conflict of interest statement

The authors declare that the research was conducted in the absence of any commercial or financial relationships that could be construed as a potential conflict of interest.
