# Supplementary material for: Transcriptomic Analysis of Staphylococcus epidermidis Biofilm-Released Cells upon Interaction with Human Blood Circulating Immune Cells and Soluble Factors
Source: Front Microbiol. 2016 Jul 21;7:1143. doi: 10.3389/fmicb.2016.01143 (PMC4955375; doi:10.3389/fmicb.2016.01143)
Supplement: Supplementary file 1 [file DataSheet1.PDF]

frontiers

Supplementary Material

Transcriptomic analysis of *Staphylococcus epidermidis* biofilm-released cells upon interaction with human blood circulating immune cells and soluble factors

Angela França*, Gerald B Pier, Manuel Vilanova, Nuno Cerca

*** Correspondence:** Angela França: afranca@deb.uminho.pt

1 Supplementary Figures and Tables

1.1 Supplementary Figures

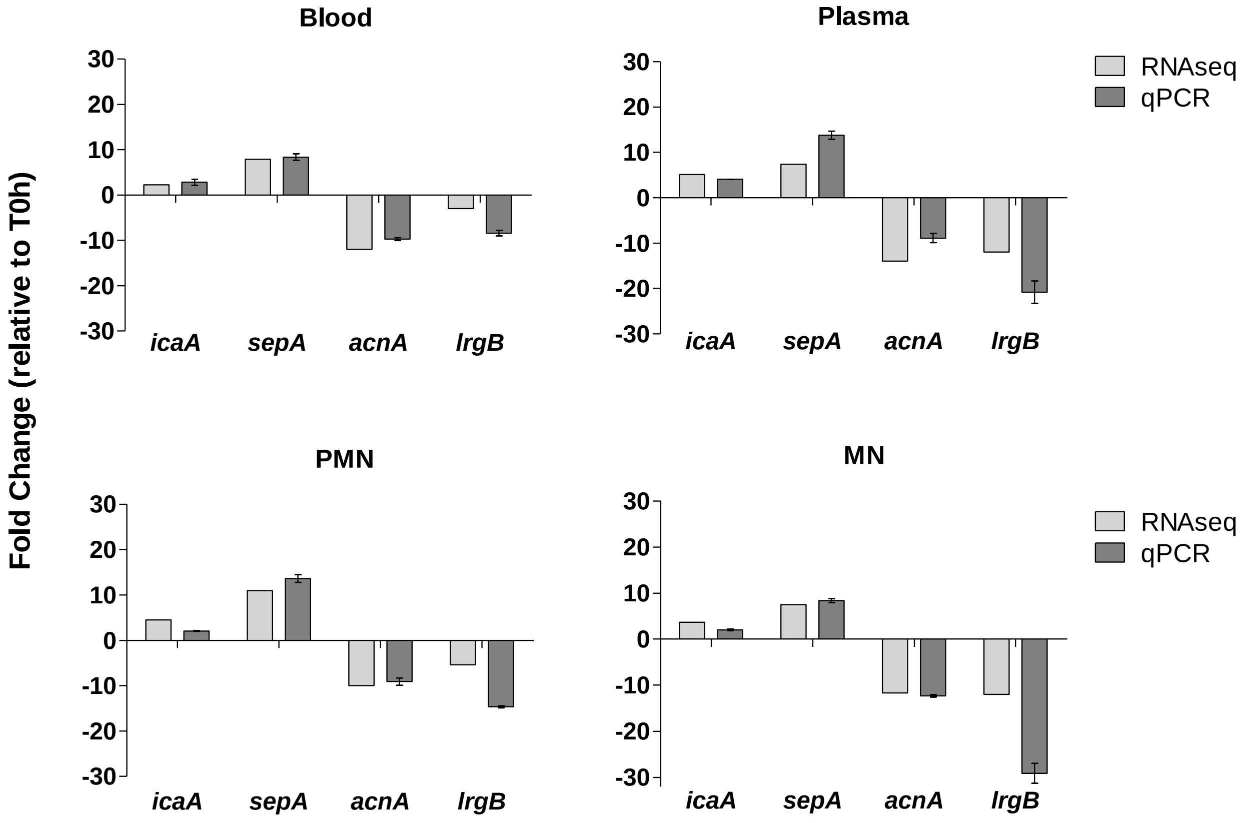

Supplementary Figure 1. RNAseq results validation by qPCR. RNA samples used to construct RNAseq libraries were tested by qPCR. The genes used for validation were randomly selected from the list of genes with significant increased or decreased transcription. The bars represent the average and the error bars the standard deviation of the qPCR technical triplicates. PMN: polymorphonuclear cells, MN: mononuclear cells, qPCR: quantitative PCR, RNAseq: RNA sequencing.

1.2 Supplementary Tables

Supplementary Table 2. Primers used for RNAseq results validation by qPCR.

Target gene		Primers sequence (5’ to 3’)	Amplicon (bp)	Efficiency (%)
***16S* rRNA**	Fw	GGGCTACACACGTGCTACAA	176	97
	Rv	GTACAAGACCCGGGAACGTA		
acnA	Fw	CATATTGGCCTACCGGAGAA	118	97.5
	Rv	TCACGAGAAGATCCCATTCC		
icaA	Fw	TGCACTCAATGAGGGAATCA	134	100
	Rv	TAACTGCGCCTAATTTTGGATT		
lrgB	Fw	ATATCGCAAGCGCGAAGTAT	165	90
	Rv	ATTGCTGTCGTTGCAGCTT		
sepA	Fw	TCTTAAGGCATCTCCGCCTA	196	97
	Rv	GTCTGGTGCGAATGATGTTG		
